# Preference for endoscopic screening of upper gastrointestinal cancer among Chinese rural residents: a discrete choice experiment

**DOI:** 10.3389/fonc.2022.917622

**Published:** 2022-07-27

**Authors:** Ruyue Liu, Youhua Lu, Yifan Li, Wenjian Wei, Chen Sun, Qianqian Zhang, Xin Wang, Jialin Wang, Nan Zhang

**Affiliations:** ^1^ School of Public Health, Weifang Medical University, Weifang, China; ^2^ Shandong Cancer Hospital and Institute, Shandong First Medical University and Shandong Academy of Medical Sciences, Jinan, China; ^3^ Cheeloo College of Medicine, Shandong University, Jinan, China; ^4^ School of Public Health, Shandong University, Jinan, China; ^5^ School of Public Health, Sun Yat-Sen University, Guangzhou, China

**Keywords:** upper gastrointestinal cancer, endoscopic screening, discrete choice experiment, preference, rural residents

## Abstract

**Background:**

The low uptake rate of upper gastrointestinal cancer (UGC) screening substantially reduces the benefits of endoscopic screening. This study aimed to obtain residents’ UGC screening preferences to optimize screening strategies and increase the participation rate.

**Methods:**

A discrete choice experiment (DCE) was conducted to assess UGC screening preferences of 1,000 rural residents aged 40 to 70 years from three countries (Linqu, Feicheng, and Dongchangfu) of Shandong province in China. The DCE questionnaire was developed from five attributes: out-of-pocket costs, screening interval, regular follow-up for precancerous lesions, mortality reduction, and screening technique. The data from the DCE were analyzed within the framework of random utility theory using a mixed logit model.

**Results:**

In total, 926 of 959 residents who responded were analyzed. The mean (SD) age was 57.32 (7.22) years. The five attributes all significantly affected residents’ preferences, and the painless endoscopy had the most important impact (*β*=2.927, P<0.01), followed by screening interval of every year (*β* = 1.184, P<0.01). Policy analyses indicated that switching the screening technique to painless endoscopy would increase the participation rate up to 89.84% (95%CI: 87.04%-92.63%). Residents aged 40–49, with a history of cancer, with a family income of more than ¥30,000 were more likely to participate in a screening.

**Conclusions:**

UGC screening implementation should consider residents’ preferences to maximize the screening participation rate. Resources permitting, we can carry out the optimal screening program with shorter screening intervals, lower out-of-pocket costs, less pain, follow-up, and higher UGC mortality reduction.

## Introduction

Upper gastrointestinal cancer (UGC), including esophageal and gastric cancer, is one of the most commonly diagnosed cancers worldwide. An estimated 16.04 million new UGC cases (8.31% of total cancers) and 13.13 million UGC deaths (13.13% of total cancer deaths) occurred in 2020 throughout the world, with China alone accounting for above 50% of cases and deaths, respectively (1). The survival rate of UGC patients largely depends on the disease stage at diagnosis. The overall 5-year survival rate of UGC patients is only about 30% when diagnosed at an advanced stage, but it can reach 90% and above if detected and treated earlier (2, 3).

Endoscopy followed by biopsy is the gold standard with high sensitivity and specificity for the diagnosis of UGC (4), which has been widely adopted in many East Asian countries, such as China, Japan, and Korea. Among these countries, Japan and Korea have implemented a national endoscopic screening for gastric cancer and achieved good results (5, 6). In China, since the implementation of population-based UGC screening in 2005, endoscopic screening for esophageal cancer and gastric cancer has been performed in more than 194 high-risk areas throughout the country, and 32,000 patients have been found, with a detection rate of 1.69% (2, 4). The current evidence from population studies has confirmed that endoscopic screening is an effective intervention method to reduce the morbidity and mortality of esophageal cancer and gastric cancer (7, 8). In addition, several economic evaluation studies (9–11) from South Korea, USA, and China showed that endoscopic screening for esophageal and gastric cancer is cost-effective compared with no screening.

However, the participation rate of the target population largely impacts the effectiveness of cancer screening (12). The endoscopic screening compliance is found to be only 48.62% in China (7). In some developed countries where population-based endoscopic screening has been going on for a long time, such as Japan and Korea, the participation rate is still lower than 50% (13, 14). This low participation rate is a huge challenge that needs to be addressed to maximize the benefits of endoscopic screening. Individuals’ screening preferences (willingness) have been shown to largely determine the UGC screening participation rate (9). Introducing preference factors into the decision-making process will improve residents’ experience and satisfaction and increase the screening compliance (15).

In recent years, discrete choice experiments (DCEs) have increasingly been used to quantify individuals’ preferences in cancer screening areas, especially in colorectal cancer, breast cancer, and cervical cancer (16–19). These data provide emphasis on the importance of DCE to obtain individuals’ preference trade-off in different screening strategies to optimize screening strategies and improve the participation rate. However, there are few DCE studies on individuals’ preferences for UGC screening, and no related study has been done in China.

This study, therefore, aims to determine individuals’ preferences and willingness to pay (WTP) on UGC screening in rural China by using a DCE and predict the participation rate of various UGC screening options to help policymakers design effective population-based screening programs.

## Materials and methods

### Discrete choice experiment

A discrete choice experiment (DCE) is a stated preference (SP) survey that explicates how people make decision by balancing product factors (e.g., characteristics of screening programs) and has been widely used in healthcare research (20, 21). In a DCE, respondents are asked to choose the most effective option among several alternative programs composed of different attribute levels (22). DCEs can figure out which characteristics (attribute levels) influence whether people choose to take a UGC screening program. In this study, we adhered to the International Society for Pharmacoeconomics and Outcomes Research (ISPOR) reporting guideline for DCE (21).

### Attributes and levels definition

The attributes and levels were selected in a stepwise manner including literature review, expert interview, and focus groups with the target population (23). Firstly, we conducted a literature review to identify attributes and levels. Attributes on screening technique, interval, cost, sensitivity, specificity, mortality reduction, follow-up, pain, waiting time for screening reports, and location of the test had a great influence on residents’ preferences of UGC screening. Since endoscopy is the gold standard for the diagnosis of UGC in China, we did not retain the attributes of sensitivity and specificity (4, 12, 14, 17, 24–30). Secondly, interviews with six experts (three clinicians specialized in UGC from Shandong Cancer Hospital, an expert who majored in public health from Shandong University, and two experts specialized in endoscopic screening from Feicheng People’s Hospital) were conducted to confirm these attributes, resulting in the following six attributes: out-of-pocket costs, time waiting for screening results, screening interval, screening technique, mortality reduction, and regular follow-up for precancerous lesions. Then, two focus-group interviews with 10 residents aged 40 to 70 from the Endoscopic Screening Department of Feicheng People’s Hospital were conducted. In the two focus groups (n = 5 × 2), they were asked to indicate which attribute of UGC screening tests they would expect to be important and to rank them in order of importance in their decision to participate in a screening program. Finally, the survey results included five attributes: out-of-pocket costs, screening interval, screening technique, mortality reduction, and regular follow-up for precancerous lesions. In addition, we identified the extreme ranges of the attribute levels from the literature review of existing UGC screenings. The selected attributes and their levels are summarized in **Table 1**.

**Table 1:** Attributes and levels for upper gastrointestinal cancer screening.

Attributes	Levels	Definitions
Out-of-pocket costs	¥0	After receiving subsidies or insurance reimbursement, individuals pay the remaining screening fees.
	¥100	
	¥300	
	¥500	
Screening interval	Every year	Frequency of the endoscopy screening in an individual’s lifetime.
	Every 2 years	
	Every 5 years	
	Once in a lifetime	
Regular follow-up for precancerous lesions	Yes	Regular follow-up is provided or not for precancerous lesions such as gastritis, intraepithelial neoplasia, and dysplasia.
	No	
Mortality reduction	15%	The extent to which an individual’s risk of death is reduced after participating in endoscopic screening.
	30%	
	45%	
	60%	
Screening technique	Endoscopy	Endoscopy or painless(anesthesia) endoscopy screening test.
	Painless (anesthesia) endoscopy	

### Questionnaire design

A total of 256 (43 × 22) possible scenarios and 32,640 ((256 × 255)/2) unique choice sets were generated by the full factorial design (31), which were not feasible to ask residents to complete. To reduce the response burden, 16 choice sets were generated and divided into two blocks using D-efficiency design with SAS9.4 software. One fixed choice set (27) (option A is definitely better than option B) was included in each block to check the residents’ understanding of the questionnaire (32). Therefore, this study had a total of two different questionnaire versions containing nine DCE choice sets. In each choice set, two hypothetical UGC screening options (option A and option B) and an opt-out option were included (see **Supplementary Table S1**). Then a pilot study was conducted, and we found that the length of the questionnaire and residents’ understanding were in line with expectations, without any significant change.

### Study population and data collection

The methods for determining the sample size varies at present (33). The most common method, which is proposed by Johnson and Orem (34, 35), is the rule of thumb: n > 500*c/(t*a), where 500 is a fixed variable, c denotes the largest number of levels for a certain attribute, a indicates the number of DCE choice sets per block of questionnaire, and t means the number of alternatives per DCE choice set (not including “Opt-out”). Accordingly, the sample size required for this study should be more than 112 respondents (500 × 4/(2 × 9) = 112). Considering the possibility of conducting further subgroup analyses, we increased the sample size to n = 1,000.

Then, we used a face-to-face interview to collect data. Based on different economic development levels, we selected 1,000 Chinese rural residents aged 40–70 years from three countries (i.e., Linqu, Feicheng, and Dongchangfu) of Shandong province in China. In the first step, residents were asked to choose the screening program that generates the highest utility to them from two hypothetical programs, and in the second step, they were asked to answer whether to undergo the screening program in real life.

### Data analysis

The out-of-pocket costs attribute was set as a continuous variable to calculate the WTP, and all other attributes were set as classification variables coded by dummy variables (36). All statistical analyses were conducted using Stata 16.0 software.

The data from the DCE were analyzed within the framework of random utility theory using a mixed logit (MIXL) model. Based on the random utility framework, the utility function can be expressed as:


$${\text{U}}_{\text{nij}}={\text{V}}_{\text{nij}}+{\text{ϵ}}_{\text{nij}}=\beta_{0}+{\text{X}}_{1ij}\beta_{1}+{\text{X}}_{2\text{ij}}\beta_{2}+\ldots +{\text{X}}_{\text{nij}}\beta_{\text{n}}+\epsilon_{\text{nij}}$$

where U_nij_ refers to the utility obtained by respondent n choosing alternatives i in choice scenario j. V_nij_ is a systematic component specified as the observable total utility, and ϵ_nij_ is the error term. X refers to the five attributes and their levels, and *β* reflects the values of each attribute and the horizontal regression coefficient (*β*_0_ represents a fixed constant term).

Importance scores for each attribute showed the contribution of each attribute relative to other attributes in decision making. Scores were calculated by dividing the maximum utility of an attribute by the total utility of all attributes.

The WTP can be estimated as the ratio of the value of the coefficient of other attribute levels to the negative of the cost attribute ( $-\frac{\beta X}{\beta M}$ ). In this context, the WTP showed the relative monetary value that respondents place on different screening characteristics, which would facilitate our understanding of the relative importance of non-monetary attributes in a DCE.

A useful output was how the probability of choosing a given screening test changes as levels of attributes are changed. To assess the expected uptake of a screening program, we applied the model as shown in the following form (37).


$$\text{Pi}=\frac{exp^{\beta Xi}}{\sum^{}exp^{\beta Xj}}$$

where x is a vector of attribute level coefficients, and *β* reflects the values of each attribute level and the horizontal regression coefficient. The attitudes of respondents to participate in a screening test are calculated by entering the constant term ASC (*β*_0_) into the model. The size of the coefficient indicates how important the attribute level is to the respondent’s choice. A positive sign implies that the attribute has a positive impact on the take-up of a given screening test; a negative coefficient is the opposite (37). In the results of a DCE, the mean coefficients reflect the relative preference weights, and the standard deviation reflects the extent of preference heterogeneity (38).

All respondents provided written informed consent. This study was approved by the Institutional Ethical Review Board of Shandong Cancer Hospital and Institute (Reference No. SDTHEC201909001).

## Results

### Respondents

Of the 1,000 invited residents, 959 (including 33 respondents who did not pass the consistency test) completed the questionnaires (response rate: 95.9%). The sensitivity analyses indicated that removing respondents who failed the rationality test did not considerably change the outcome (see **Supplementary Table S2**). Considering the accuracy of the results, 926 respondents who passed the consistency test were finally included for preference estimation by the mixed logit model. **Table 2** summarizes the demographic of the 926 respondents. The mean age (SD) was 57.32 (7.22 years), and 66% of the respondents were women. A family history of cancers was reported by 19.76% of the respondents, and 434 respondents’ annual family income was lower than ¥10,000. No statistical differences between respondents and non-respondents were found.

**Table 2:** Characteristics of the respondents.

Characteristics	Respondents		Non-respondents		*χ^2^*	*P* value
	n = 926 (who passed the consistency test)		n = 33 (who failed the consistency test)			
	n	No. (%)	n	No. (%)		
Mean age, years (SD)	57.32 (7.22)		57.36 (7.17)		−	−
Gender						
Male	315	34.02	12	36.36	0.078	0.780
Female	611	65.98	21	63.64
Age						
40-49	139	15.01	1	3.03	4.308^a^	0.095
50-59	414	44.71	19	57.58
60-70	373	40.28	13	39.39
Marital status^b^						
With a partner	888	95.90	30	90.91	1.937^a^	0.163
Without a partner	38	4.10	3	9.01
Annual family income (RMB)						
<10,000	434	46.87	21	63.64	3.917	0.141
10,000-29,999	294	31.75	6	18.18
≥30,000	198	21.38	6	18.18
Location ^c^						
Linqu	322	34.77	11	33.33	0.328	0.849
Feicheng	310	33.48	10	30.31
Dongchangfu	294	31.75	12	36.36
Family history of cancer ^d^						
Yes	183	19.76	3	9.09	2.321	0.128
No	743	80.24	30	90.91
Screening for cancer						
Ever	520	56.16	11	33.33	6.716	0.010
Never	406	43.84	22	66.67

:^a^Fisher exact probability method. ^b^Marital status: with a partner, reflecting that the individual is married and the spouse is alive; without a partner, including single, divorced, widowed. ^c^The per capita GDP in 2020 in Linqu, Feicheng, and Dongchangfu were ¥39,910, ¥80,696, and ¥50,726, respectively. ^d^History of cancer in blood relatives, including parents, grandparents, siblings, uncles, aunts, cousins. RMB, the average exchange rate between US$ and RMB in 2021 was US$1 = RMB 6.45; SD, standard deviation.

### Preferences estimates

**Table 3** shows the results of the final preferences model. The participants preferred the UGC screening over no screening (*β* = -6.829; (95%CI, -8.238 to -5.419)). All five attributes had statistical effects on residents, and the direction was consistent with our expectations. The screening technique had the most important impact on respondents, followed by out-of-pocket costs and screening interval (see **Supplementary Figure S1**). In all attribute levels, most of the residents preferred painless endoscopy (*β* = 2.927; (95%CI, 2.638 to 3.217)). Overall, the respondents preferred a screening program that has a shorter screening interval, causes less pain, has follow-up, pays lower costs, and results in a higher decrease in UGC-related mortality.

**Table 3:** Preference and WTP results of a mixed logit model.

Attributes and levels	Mean (preference)			SD (preference)			WTP		
	*β* (SE)	*Z value*	95% CI	*β* (SE)	*Z value*	95% CI	*β* (SE)	*Z value*	95% CI
ASC (Opt-out)	-6.829** (0.719)	-9.490	(-8.238, -5.419)	6.826** (0.569)	12.000	(5.711, 7.941)	−	−	−
Screening interval									
Once in a lifetime (Ref)									
Every year	1.184** (0.087)	13.560	(1.013, 1.355)	0.645** (0.148)	4.370	(0.356, 0.934)	277.48** (22.624)	12.260	(233.14, 321.82)
Every 2 years	1.122** (0.097)	11.600	(0.933, 1.132)	0.392 (0.261)	1.500	(-0.120,0.905)	263.01** (24.600)	10.690	(214.79, 311.22)
Every 5 years	0.971** (0.105)	9.220	(0.764, 1.177)	0.687** (0.218)	3.150	(0.260, 1.115)	227.50** (23.126)	9.840	(182.17, 272.82)
Regular follow-up for precancerous lesions									
Yes (Ref)									
No	-0.243** (0.050)	-4.820	(-0.342, -0.144)	0.010 (0.106)	0.090	(-0.198, 0.218)	-57.02** (11.959)	-4.770	(-80.46, -33.58)
Mortality reduction									
15% (Ref)									
30%	0.068 (0.089)	0.760	(-0.107, 0.243)	0.013 (0.123)	0.100	(-0.254, 0.229)	15.96 (20.981)	0.760	(25.16, 57.08)
45%	0.225* (0.103)	2.170	(0.022, 0.427)	0.010 (0.406)	0.020	(-0.806, 0.786)	52.63* (24.562)	2.140	(4.49, 100.77)
60%	0.191* (0.082)	2.330	(0.030, 0.352)	0.411* (0.199)	2.060	(0.020, 0.801)	44.81* (19.471)	2.300	(6.65, 82.97)
Screening technique									
Endoscopy (Ref)									
Painless (anesthesia) endoscopy	2.927** (0.148)	19.810	(2.638, 3.217)	2.245** (0.132)	17.010	(1.987, 2.504)	686.01** (39.843)	17.22	(607.92, 764.10)
Out-of-pocket costs	-0.004** (0.000)	-16.010	(-0.005, -0.004)	0.003** (0.000)	11.300	(0.003, 0.004)	−	−	−
Sample	926								

^*^ P< 0.05, ^* *^ P< 0.01; ASC (Opt-out), a specific constant item for opt-out; Ref, reference, which reflects a reference level in each attribute; β, which reflects the values of each attribute level and the horizontal regression coefficient; WTP, willingness to pay, which reflects residents’ willingness to pay for a certain screening program; SD, standard deviation; SE, standard error; 95% CI, 95% confidence interval.

### Willingness to pay

The results of WTP are shown in **Table 3**. When converting endoscopy to painless endoscopy, the respondents were willing to pay ¥686.01 (95% CI, 607.92 to 764.10). Compared with screening once in a lifetime, they were willing to pay about ¥250 for every year, every 2 years, and every 5 years. Theoretically, if regular follow-up was not provided, the respondents should be compensated ¥57.02 (95% CI, -80.46 to -33.58).

### Subgroup analyses

The results of MIXL and WTP among different subgroups are shown in **Table 4** (see **Supplementary Table S3**-**6**, which shows more details). Apart from the screening technique and screening interval attributes, the preferences for other attributes between different subgroups were relatively similar. Residents who came from Linqu placed more value on the screening interval attribute, and they were willing to pay ¥308.87 (95% CI, 215.64 to 402.10) to shorten the intervals from once in a lifetime to every 2 years. Compared with residents whose family income was less than ¥10,000, residents with a family income of more than ¥30,000 were willing to pay ¥681.23 (95% CI, 457.06 to 905.40) for the screening interval of every year. In comparison to other age groups, residents aged 40 to 49 favored a painless endoscopy approach. In addition, women preferred to participate in a screening test with painless endoscopy than men (*β*: 3.088 vs. 2.734, P< 0.01).

**Table 4:** The results of different subgroup analysis and WTP(*β*(WTP)).

Attributes	Location			Age			Gender		Annual family income (RMB)		
	Linqu (n=322)	Feicheng (n=310)	Dongchangfu (n=294)	40-49(n=139)	50-59(n=414)	60-69(n=373)	Male(n=315)	Female(n=611)	<10000(n=434)	10000-29999(n=294)	≥30000(n=198)
ASC (Opt-out)	-3.717^**^	-6.304^**^	-7.375^**^	-5.512^**^	-6.826^**^	-8.728^**^	-5.529^**^	-8.062^**^	-7.501^**^	-9.067^**^	-3.611^**^
Screening interval											
Once in a lifetime (Ref)											
Every year	1.487^**^ (267.34)	1.155^**^ (303.94)	1.023^**^ (237.85)	2.032^**^ (526.82)	1.456^**^ (356.49)	0.754^**^ (149.46)	1.341^**^ (323.94)	1.119^**^ (252.74)	0.875^**^ (152.10)	1.166^**^ (315.01)	2.201^**^ (681.23)
Every 2 years	1.718^**^ (308.87)	1.153^**^ (303.57)	0.604^**^ (140.42)	2.131^**^ (552.69)	1.336^**^ (327.13)	0.681^**^ (135.01)	1.329^**^ (321.12)	1.028^**^ (232.02)	0.838^**^ (145.63)	1.143^**^ (309.83)	1.979^**^ (612.61)
Every 5 years	1.089^**^ (195.74)	1.007^**^ (265.15)	0.790^**^ (183.75)	1.924^**^ (498.93)	0.873^**^ (213.69)	0.854^**^ (169.25)	0.978^**^ (236.37)	0.964^**^ (217.58)	0.823^**^ (142.96)	0.925^**^ (251.22)	1.657^**^ (512.92)
Regular follow-up for precancerous lesions											
Yes (Ref)											
No	-0.254^*^ (-45.65)	-0.256^**^ (-67.48)	-0.285^*^ (-66.19)	-0.096 (-24.77)	-0.329^**^ (-80.56)	-0.231^**^ (-45.87)	-0.244^**^ (-58.99)	-0.239^**^ (-53.88)	-0.276^**^ (-47.94)	-0.341^**^ (-90.97)	-0.087 (-27.06)
Mortality reduction											
15% (Ref)											
30%	0.015 (2.74)	0.153 (40.29)	0.024 (5.54)	0.162 (42.024)	0.059 (14.48)	0.075 (14.94)	0.103 (25.00)	0.072 (16.26)	0.215 (37.37)	0.063 (20.19)	-0.241 (-74.47)
45%	0.151 (27.16)	0.161 (42.34)	0.431^*^ (100.27)	0.453 (117.58)	0.292 (71.40)	0.141 (27.95)	0.333 (80.40)	0.182 (41.09)	0.359^*^ (62.36)	0.224 (61.57)	-0.022 (-6.91)
60%	0.052 (9.35)	0.411 (108.29)	0.077^**^ (18.01)	0.367 (95.11)	0.186 (45.53)	0.144 (28.47)	0.400^**^ (96.64)	0.086 (19.48)	0.049 (8.57)	0.360^**^ (99.18)	0.275 (85.08)
Screening technique											
Endoscopy (Ref)											
Painless (anesthesia) endoscopy	4.542^**^ (816.49)	2.078 (547.06)	3.037^**^ (706.21)	3.439^**^ (891.75)	3.046^**^ (745.75)	3.013^**^ (597.46)	2.734^**^ (660.62)	3.088^**^ (697.09)	3.217^**^ (559.08)	2.707^**^ (732.14)	3.445^**^ (1066.26)
Out-of-pocket costs	-0.006^**^	-0.004	-0.004^**^	-0.004^**^	-0.004^**^	-0.005^**^	-0.004^**^	-0.004^**^	-0.006^**^	-0.004^**^	-0.003^**^

^*^ P< 0.05, ^* *^ P< 0.01. A separate regression is run for each subgroup. The baseline job is the same in all regressions. ^a^ The per capita GDP in 2020 in Linqu, Feicheng, and Dongchangfu were ¥39,910, ¥80,696, and ¥50,726, respectively. ASC (Opt-out), a specific constant item for opt-out; Ref, reference, which reflects a reference level in each attribute; β, which reflects the values of each attribute level and the horizontal regression coefficient; WTP, willingness to pay, which reflects residents’ willingness to pay for a certain screening program; 95% CI, 95% confidence interval; RMB, US$ 1 = RMB 6.45.

### Expected UGC screening uptake

**Figure 1:**
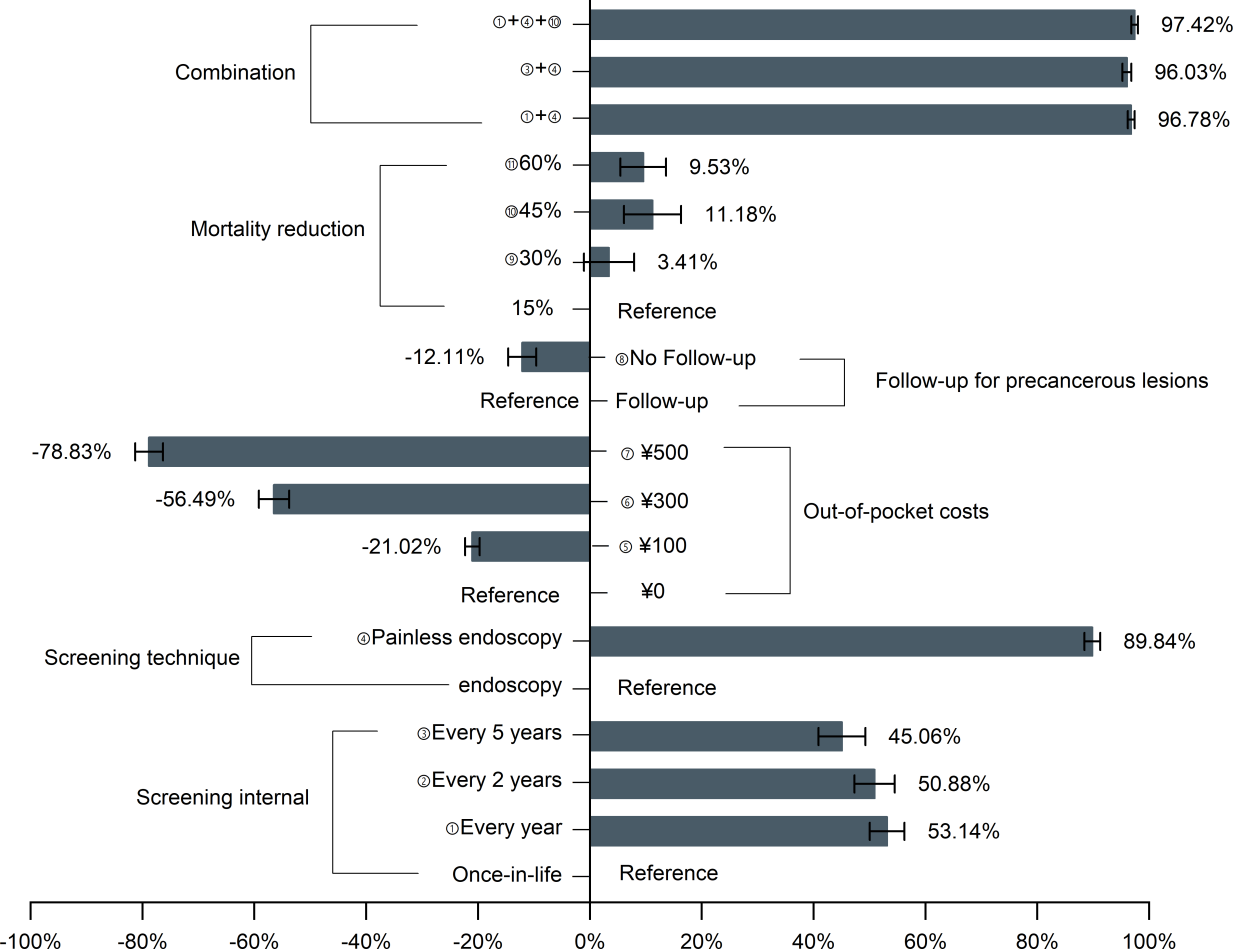
Effects of changing the screening program characteristics on the probability of participation in upper gastrointestinal cancer screening. UGC, upper gastrointestinal cancer.

**Figure 1** depicts the probability of respondents as attributes and levels were changed at the baseline level (baseline: ¥0, once in a lifetime, with follow-up, a 15% mortality reduction, and endoscopy). Increased out-of-pocket costs from ¥0 to ¥500 resulted in a 78.8% drop in participation, but changing the screening technique from endoscopy to painless endoscopy would raise participation to 89.84%. Under the optimal screening scenario (i.e., free, 1-yearly, with follow-up, 45% mortality reduction, and painless endoscopy), the participation rate of endoscopic screening would increase to 97.42%.

**Figure 2:**
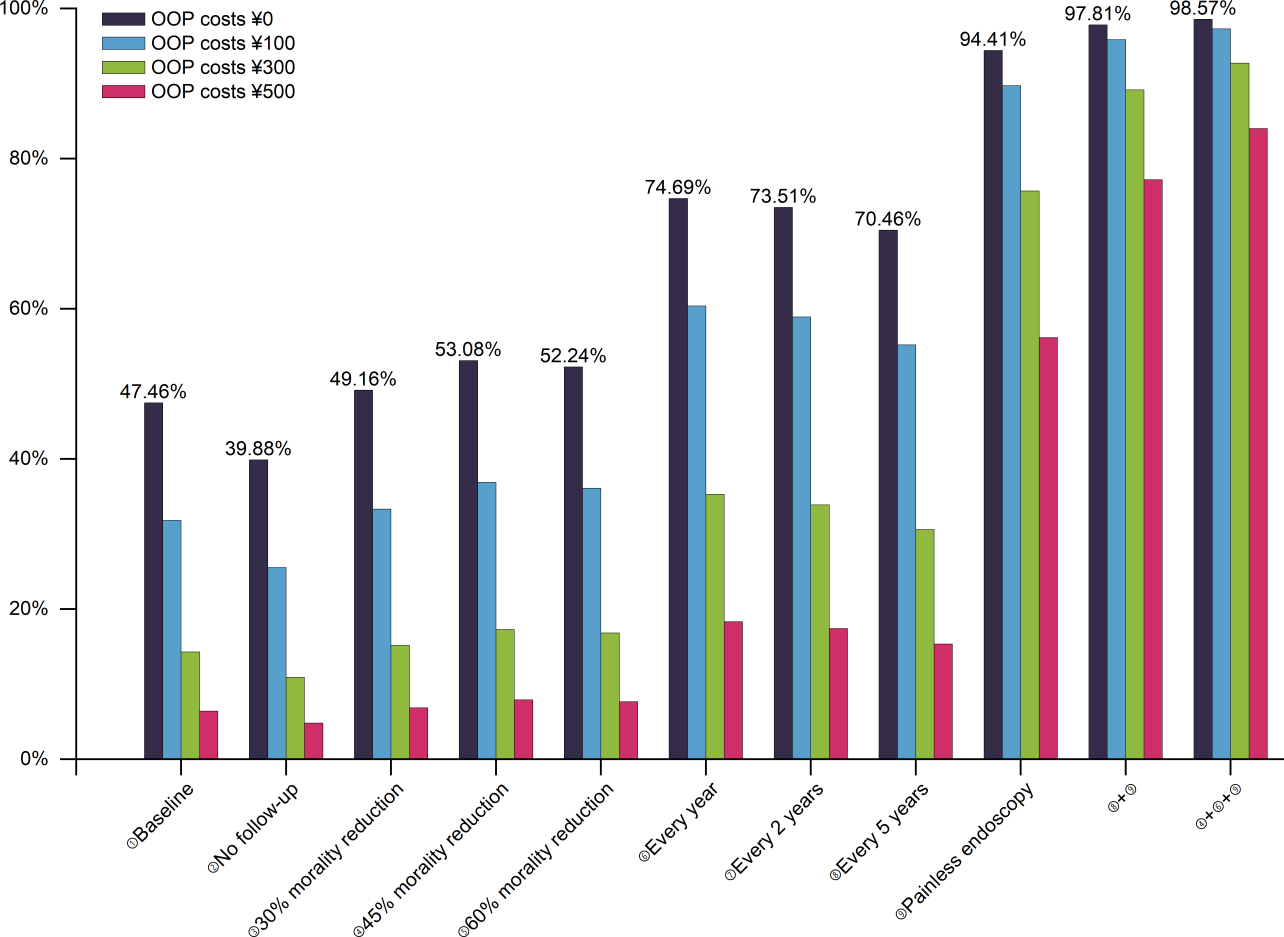
The participation rates for UGC screening at different levels of out-of-pocket costs. Baseline: once in a lifetime, with follow-up, a 15% mortality reduction, and endoscopy; UGC, upper gastrointestinal cancer; OOP, out-of-pocket.

Furthermore, the participation rates at different out-of-pocket cost levels were as shown in **Figure 2**. The initial participation rate of out-of-pocket cost of ¥0 is 47.46%, whereas the participation rate of ¥500 was only 6.42%. However, with the exception of ¥500, the participation rate of other cost levels achieved above 90% based on the ideal screening scenario combination.

## Discussion

To our knowledge, this was the first DCE study to explore the UGC screening preferences of rural residents in China. The results were consistent with our assumptions based on the current screening status in China. All five attributes considered in our study were found to be statistically significant. Rural residents preferred screening programs with a higher decreased UGC-related mortality, shorter intervals, follow-up, less pain, and lower costs.

In our study, respondents were mostly driven by painless endoscopy (WTP = ¥686.01). The respondents’ strong preference for painless endoscopy largely reflected their pursuit of comfort in the screening process. In the Netherlands, two DCEs for esophageal cancer screening showed that the participants equally preferred a screening program with less pain and discomfort (12, 39). Other studies for colorectal cancer screening concluded that the respondents preferred a no-pain or mild-pain screening program (17, 22, 40–42). The target population may ignore the improvement of other attributes in pursuit of a more comfortable screening experience. Thus, it was an effective means to improve the participation rate of UGC screening by advocating the painless endoscopy.

A previous DCE study on Barrett’s esophagus concluded that surveillance every 5 years would lead to a 26% reduction, while surveillance every 3 to 3.5 years would result in a 7% increase in participation (30). The subjects preferred endoscopic screening every 1–2 years compared to a longer screening interval (43). Similar to the above studies, the subjects in our study preferred a shorter screening interval, in which the screening interval of every year is the most popular. However, it may be unrealistic to establish an annual screening interval in some countries with limited resources. We further found that raising the screening interval from once-lifetime to every 5 years can increase the probability by 45.06%, but increasing it from every 5 years to 2 years increases the probability by only 5.82%. Clearly, when the screening interval was shortened to every 5 years, the increase in participation rate slowed down apparently. Existing studies have also shown that every 5 years or a longer screening interval were more popular among the population (17, 18).

Our study also revealed the heterogeneity of population preferences in different subgroups. Consistent with the findings of Peter et al. (12), women preferred a screening program with less pain, and men preferred a lower risk of death. The subjects of different ages had significant differences in preferences for cancer screening and treatment (24, 44). Residents aged 40–49 years with a higher family income had a higher WTP for screening, and they seemed to have a higher demand and better compliance for UGC screening.

A useful output when using DCEs was how the probability of choosing a given option changes with attribute levels (37). We found that the 47.46% participation rate of current screening strategies was very close to the actual uptake rates of 48.62% and 49% (7, 10), indicating that the predicted results in this study were accurate and dependable. We also found that the UGC screening strategies with follow-up, shorter interval, less pain, higher mortality reduction, and lower costs had a higher participation rate. However, as UGC screening strategies, in addition to the participation rate, the cost of health economics also needs to be considered. Xia et al. (10) noted that an endoscopic screening for EC and GC would be more cost-effective than no screening regardless of the initial screening age or screening interval. This finding is consistent with the conclusion reported in our previous research, and the selected attributes and levels in this study draw on the dominance strategies screened from our previous work. It can be considered that the screening strategies consisting of different attributes and levels in this study are cost-effective and feasible under the level of per capita GDP to screen high-risk groups aged 40–70 in China. Nonetheless, further studies are still needed to validate the economics of the UGC screening strategies proposed in this study.

One study pointed out that the cost was always the most important attribute in colorectal cancer screening, and respondents preferred a lower cost (22). Another study on esophageal cancer screening also indicated that the participation rate would decrease by 48% if subjects were required to pay $500 (30). In line with their findings, we discovered that as out-of-pocket costs rise, participation rates decline dramatically. Nonetheless, by optimizing combinations of attributes and levels, the high-cost screening program can be made more appealing. Further analysis showed that the participation rate of out-of-pocket costs of ¥100 and ¥0 was similar when converting an endoscopy to a painless endoscopy. Even if residents have to pay ¥500 in the optimal screening scenario, their participation rate still reached over 80%. However, there were few studies on cancer screening financing mechanisms and user out-of-pocket cost levels in China. Moreover, how to ensure the participation rate of users when paying needs further empirical research.

Contrary to other studies (17, 40, 42), the mortality reduction in this study showed an insignificant effect, and the 30% reduction of death risk was not statistically significant. There were probably two reasons. First, all respondents in this study were rural residents aged 40–70, and they were far less concerned about reducing the risk of cancer deaths than younger generations. Second, the respondents did not understand the concept of death risk at all. Nevertheless, only a minority (3.44%) of respondents failed the test of rationality, suggesting that most respondents understand the DCE questions. In addition, previous studies on colorectal cancer screening preference concluded that characteristics associated with accuracy (sensitivity and specificity) seemed to be more important than those related to screening procedures (45–47). However, the accuracy attribute was not used in this study since endoscopy as the gold standard of UGC screening (an accuracy rate of more than 95%) had been used widely in China.

This study is characterized by several strengths. First, our sample size of 1,000 was substantially larger than the recommended at least 20 respondents per version by the DCE User’s Guide (37), which makes our results more representative and sufficient to provide references for other countries. In addition, an opt-out option was included, since it better reflects actual screening participation and prevents overestimation of screening uptake. Finally, this is the first DCE study on UGC endoscopic screening in a developing country. In a global context, these results are important for optimizing future endoscopic screening strategies in light of the impact of the high morbidity and mortality of UGC, especially in developing nations.

Several limitations need to be discussed. First, only five attributes were included in the DCE to simplify the choice tasks. Thus, not all aspects of the UGC screening test were captured in this DCE. In the design stage, we conducted a literature review, expert interviews, and two focus groups to identify the five most important attributes that can explain the target population’s preference to the greatest extent. Second, this study is a stated preference, which may be different from revealed preferences (48). The revealed preferences should be examined after implementing the UGC screening programs. Third, our results showed that residents preferred a 45% reduction in death risk than 60%. In the context of this study, it is difficult for us to determine exactly whether the phenomenon is due to the rural residents’ lack of understanding or they do not care about morality reduction, and further research is needed to explore.

In conclusion, residents were positive for UGC screening, and their participation rate was greatly affected by the implementation of painless endoscopy. For maximizing the population uptake rate, an optimal UGC endoscopic screening with shorter screening intervals, lower out-of-pocket costs, painlessness, follow-up, and higher UGC mortality reduction should be implemented according to resources’ availability. Our data provided some insights for clinicians and policymakers to develop screening programs with a higher population uptake.

## Data availability statement

The raw data supporting the conclusions of this article will be made available by the authors, without undue reservation.

## Ethics statement

This study was approved by the Institutional Ethical Review Board of Shandong Cancer Hospital and Institute (Reference No. SDTHEC201909001). The patients/participants provided their written informed consent to participate in this study.

## Author contributions

RL, study concept and design, data collection, data entry, data interpretation, and statistical analysis, drafting of manuscript, and critical revision of the manuscript. YHL, critical revision of the manuscript. YFL and WW, data collection, data entry, and suggestion with revision of the manuscript. CS, QZ, XW, and JW, suggestion with revision of the manuscript. NZ, study concept and design, residents’ recruitment, data interpretation, and critical revision of the manuscript. All authors contributed to the article and approved the submitted version.

## Funding

This research was funded by the National Natural Science Foundation of China, grant number 71904109.

## Acknowledgments

The authors wish to thank all of the residents, collaborators, and graduate students who participated in the field survey and discrete choice experiment study, as well as the experts and work staff in sample regions who assisted with participant recruitment and data collection.

## Conflict of interest

The authors declare that the research was conducted in the absence of any commercial or financial relationships that could be construed as a potential conflict of interest.

## Publisher’s note

All claims expressed in this article are solely those of the authors and do not necessarily represent those of their affiliated organizations, or those of the publisher, the editors and the reviewers. Any product that may be evaluated in this article, or claim that may be made by its manufacturer, is not guaranteed or endorsed by the publisher.
