# Supplementary material for: Preference for endoscopic screening of upper gastrointestinal cancer among Chinese rural residents: a discrete choice experiment
Source: Front Oncol. 2022 Jul 27;12:917622. doi: 10.3389/fonc.2022.917622 (PMC9363665; doi:10.3389/fonc.2022.917622)
Supplement: Supplementary file 1 [file DataSheet_1.docx]

Supplementary Material

# Supplementary Figures and Tables

## Supplementary Figures
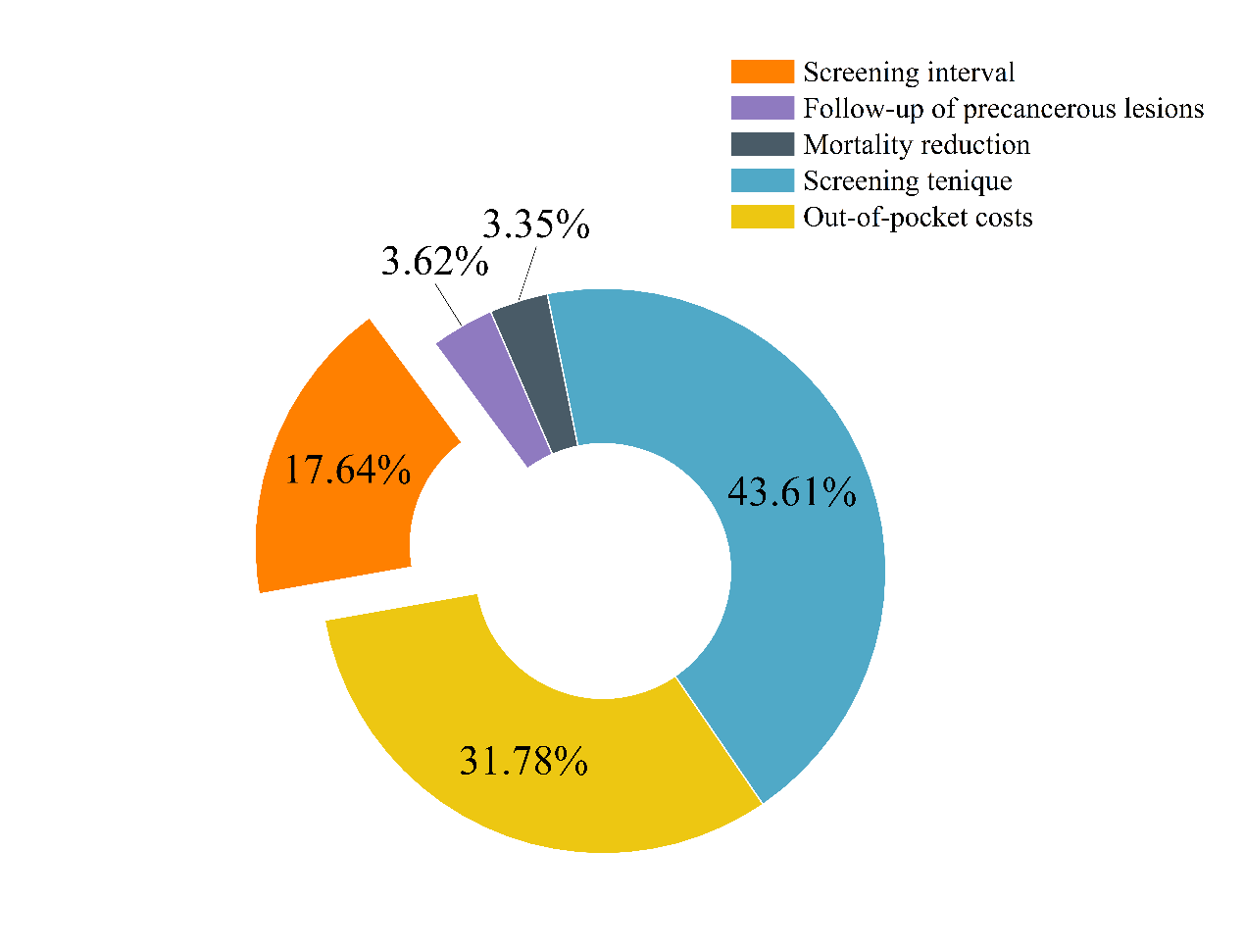
Supplementary Figure S1. Relative importance score of different attributes

## Supplementary Tables

**Supplementary Table S1.** Example of DCE choice sets

| **Attributes** | **Option A** | **Option B** |
| --- | --- | --- |
| Out-of-pocket costs | ¥100 | ¥300 |
| Screening interval | Every 2 years | Every year |
| Regular follow-up for precancerous lesions | Yes | No |
| Mortality reduction | 15% | 45% |
| Screening technique | Endoscopy | Painless (anesthesia) endoscopy |
| **Which of these options would you prefer?** | □ | □ |
| **Would you choose to be screened in a real life?** | Yes | No |

**Supplementary Table S2.** Sensitivity analysis

| **Attributes and levels** | **Pass the consistency test（n=926）** | | | | **Failed the consistency test（=959）** | | | | |
| --- | --- | --- | --- | --- | --- | --- | --- | --- | --- |
|  | ***β* coefficient (SE)** | ***P* value** | **95%CI** | | ***β* coefficient (SE)** | | ***P* value** | **95%CI** | |
| ASC (Opt-out) | -6.829 (0.719) | 0.000 | -8.238 | -5.419 | | -7.004 (0.676) | 0.000 | -8.329 | -5.680 |
| Screening interval | | | | | | | | | |
| Once in a lifetime (Ref) | | | | | | | | | |
| Every year | 1.184 (0.087) | 0.000 | 1.013 | 1.355 | | 1.138 (0.086) | 0.000 | 0.969 | 1.307 |
| Every 2 years | 1.122 (0.097) | 0.000 | 0.933 | 1.312 | | 1.065 (0.096) | 0.000 | 0.877 | 1.252 |
| Every 5 years | 0.971 (0.105) | 0.000 | 0.764 | 1.177 | | 0.957 (0.104) | 0.000 | 0.753 | 1.161 |
| Regular follow-up for precancerous lesions | | | | | | | | | |
| Yes (Ref) |  |  |  |  | |  |  |  |  |
| No | -0.243 (0.050) | 0.000 | -0.342 | -0.144 | | -0.221 (0.050) | 0.000 | -0.319 | -0.122 |
| Mortality reduction | | | | | | | | | |
| 15% (Ref) |  |  |  |  | |  |  |  |  |
| 30% | 0.068 (0.089) | 0.445 | -0.107 | 0.243 | | 0.070 (0.088) | 0.423 | -0.102 | 0.242 |
| 45% | 0.225 (0.103) | 0.030 | 0.022 | 0.427 | | 0.209 (0.101) | 0.040 | 0.010 | 0.408 |
| 60% | 0.191 (0.082) | 0.020 | 0.030 | 0.352 | | 0.201(0.081) | 0.013 | 0.042 | 0.359 |
| Screening technique | | | | | | | | | |
| Endoscopy (Ref) | | | | | | | | | |
| Painless (anesthesia) endoscopy | 2.927 (0.148) | 0.000 | 2.638 | 3.217 | | 2.880 (0.151) | 0.000 | 2.584 | 3.177 |
| Out-of-pocket costs | -0.004 (0.000) | 0.000 | -0.005 | -0.004 | | -0.004 (0.000) | 0.000 | -0.005 | -0.004 |

Note: * P <0.05, * * P <0.01; ASC (Opt-out)：a specific constant item for opt-out; Ref: reference, which reflects a reference level in each attribute; β coefficient: which reflects the values of each attribute level and the horizontal regression coefficient; SE, standard error; 95% CI, 95% confidence interval.

**Supplementary Table S3.** Preference and WTP of residents in different regions

| **Attributes and levels** | **Linqu (n=322)** | | | **Feicheng (n=310)** | | | **Dongchangfu (n=294)** | | |
| --- | --- | --- | --- | --- | --- | --- | --- | --- | --- |
|  | ***β* coefficient**  **(95% CI)** | ***Z* value** | **WTP**  **(95% CI)** | ***β* coefficient**  **(95% CI)** | ***Z* value** | **WTP**  **(95% CI)** | ***β* coefficient**  **(95% CI)** | ***Z* value** | **WTP**  **(95% CI)** |
| ASC (Opt-out) | -3.717  (-5.685,-1.748) | -3.700^**^ |  | -6.304  (-8.175,-4.434) | -6.610^**^ |  | -7.375  (-9.951,-4.799) | -5.610^**^ |  |
| Screening interval | | | | | | | | | |
| Once in a lifetime (Ref) | | | | | | | | | |
| Every year | 1.487  (1.072,1.903) | 7.010^**^ | 267.34  (189.27,345.39) | 1.155  (0.918,1.392) | 9.550^**^ | 303.94  (231.11,376.77) | 1.023  (0.716,1.329) | 6.540^**^ | 237.85  (161.00,314.69) |
| Every 2 years | 1.718  (1.225,2.212) | 6.820^**^ | 308.87  (215.64,402.10) | 1.153  (0.881,1.426) | 8.290^**^ | 303.57  (223.16,383.99) | 0.604  (0.288,0.920) | 3.740^**^ | 140.42  (63.42,217.41) |
| Every 5 years | 1.089  (0.603,1.574) | 4.390^**^ | 195.74  (112.68,278.79) | 1.007  (0.718,1.296) | 6.830^**^ | 265.15  (192.84,337.47) | 0.790  (0.438,1.142) | 4.400^**^ | 183.75  (106.46,261.03) |
| Regular follow-up for precancerous lesions | | | | | | | | | |
| Yes (Ref) | | | | | | | | | |
| No | -0.254  (-0.491,-0.017) | -2.100^*^ | -45.65  (-87.91,-3.40) | -0.256  (-0.394,-0.119) | -3.650^**^ | -67.48  (-105.51,-29.45) | -0.285  (-0.461,-0.108) | -3.160^*^ | -66.19  (-107.92,-24.46) |
| Morality reduction | | | | | | | | | |
| 15% (Ref) | | | | | | | | | |
| 30% | 0.015  (-0.396,0.426) | 0.070 | 2.74  (-71.17,76.65) | 0.153  (-0.096,0.402) | 1.210 | 40.29  (-26.15,106.74) | 0.024  (-0.286,0.334) | 0.150 | 5.54  (-66.70,77.79) |
| 45% | 0.151  (-0.322,0.624) | 0.630 | 27.16  (-58.22,112.53) | 0.161  (-0.133,0.454) | 1.070 | 42.34  (-36.17,120.86) | 0.431  (0.069,0.794) | 2.330^*^ | 100.27  (13.42,187.12) |
| 60% | 0.052  (-0.300,0.404) | 0.290 | 9.35  (-54.00,72.70) | 0.411  (0.171,0.652) | 3.360^**^ | 108.29  (41.43,175.15) | 0.077  (-0.202,0.356) | 0.540 | 18.01  (-47.32,83.33) |
| Screening technique | | | | | | | | | |
| Endoscopy (Ref) | | | | | | | | | |
| Painless (anesthesia) endoscopy | 4.542  (3.675,5.409) | 10.270^**^ | 816.49  (647.86,985.12) | 2.078  (1.789,2.368) | 14.060^**^ | 547.06  (447.13,646.99) | 3.037  (2.491,3.582) | 10.920^**^ | 706.21  (564.28,848.14) |
| Out-of-pocket costs | -0.006  (-0.007,-0.004) | -8.640^**^ | - | -0.004  (-0.004,-0.003) | -10.860^**^ |  | -0.004  (-0.005,-0.003) | -8.060^**^ | - |

Note: ^*^ P <0.05, ^* *^ P <0.01; The per capita GDP in 2020 in Linqu, Feicheng and Dongchangfu were ¥39,910, ¥80,696 and¥ 50,726, respectively; ASC (Opt-out)：a specific constant item for opt-out; Ref: reference, which reflects a reference level in each attribute; *β* coefficient: which reflects the values of each attribute level and the horizontal regression coefficient; WTP: willingness to pay, which reflects residents’ willingness to pay for a certain screening program; 95% CI, 95% confidence interval.

**Supplementary Table S4.** Preference and WTP of residents in different ages (year)

| **Attributes and levels** | **40-49（n=139）** | | | **50-59（n=414）** | | | **60-69（n=373）** | | | |
| --- | --- | --- | --- | --- | --- | --- | --- | --- | --- | --- |
|  | ***β* coefficient**  **(95% CI)** | ***Z* value** | **WTP**  **(95% CI)** | ***β* coefficient**  **(95% CI)** | ***Z* value** | **WTP**  **(95% CI)** | ***β* coefficient**  **(95% CI)** | ***Z* value** | **WTP**  **(95% CI)** | |
| ASC(Opt-out) | -5.512  (-8.376,-2.648) | -3.770^**^ | *-* | -6.826  (-8.902,-4.750) | -6.440^**^ | - | -8.728  (-11.55,-5.900) | -6.050^**^ | - | |
| Screening interval | | | | | | | | | | |
| Once in a lifetime (Ref) | | | | | | | | | | |
| Every year | 2.032  (1.377,2.686) | 6.090^**^ | 526.82  (326.69,726.94) | 1.456  (1.178,1.734) | 10.260^**^ | 356.49  (274.08,438.91) | 0.754  (0.506,1.002) | 5.950^**^ | 149.46  (98.76,200.16) | |
| Every 2 years | 2.131  (1.429,2.834) | 5.950^**^ | 552.69  (347.66,757.73) | 1.336  (1.021,1.651) | 8.310^**^ | 327.13  (239.36,414.89) | 0.681  (0.395,0.967) | 4.670^**^ | 135.01  (77.86,192.16) | |
| Every 5 years | 1.924  (1.155,2.693) | 4.900^**^ | 498.93  (311.03,686.83) | 0.873  (0.568,1.178) | 5.610^**^ | 213.69  (142.12,285.26) | 0.854  (0.534,1.173) | 5.240^**^ | 169.25  (110.9,227.51) | |
| Regular follow-up for precancerous lesions | | | | | | | | | | |
| Yes (Ref) | | | | | | | | | | |
| No | -0.096  (-0.368,0.177) | -0.690 | -24.77  (-96.47,46.92) | -0.329  (-0.488,-0.170) | -4.060^**^ | -80.56  (-120.42,-40.70) | -0.231  (-0.386,-0.077) | -2.940^**^ | -45.87  (-77.25,-14.50) | |
| Mortality reduction | | | | | | | | | | |
| 15% (Ref) | | | | | | | | | | |
| 30% | 0.162  (-0.345,0.669) | 0.630 | 42.024  (-91.28,175.33) | 0.059  (-0.205,0.323) | 0.440 | 14.48  (-50.49,79.45) | 0.075  (-0.209,0.359) | 0.520 | 14.94  (-41.46,71.34) | |
| 45% | 0.453  (-0.143,1.050) | 1.490 | 117.58  (-45.94, 281.09) | 0.292  (-0.019,0.603) | 1.840 | 71.40  (-6.74,149.53) | 0.141  (-0.186,0.468) | 0.850 | 27.95  (-37.19,93.09) | |
| 60% | 0.367  (-0.072,0.805) | 1.640 | 95.11  (-24.87,215.08) | 0.186  (-0.073,0.445) | 1.410 | 45.53  (-18.60,109.65) | 0.144  (-0.112,0.400) | 1.100 | 28.47  (-22.85,79.80) | |
| Screening technique | | | | | | | | | | |
| Endoscopy (Ref) | | | | | | | | | | |
| Painless (anesthesia) endoscopy | 3.439  (2.454,4.424) | 6.850^**^ | 891.75  (595.14,1188.36) | 3.046  (2.608,3.484) | 13.630^**^ | 745.75  (605.72,885.77) | 3.013  (2.555,3.472) | 12.880^**^ | 597.46  (502.3,692.59) | |
| Out-of-pocket costs | -0.004  (-0.005, -0.002) | -5.470^**^ |  | -0.004  (-0.005,-0.003) | -10.090^**^ |  | -0.005  (-0.006,-0.004) | -11.160^**^ |  | |
|  | | | | | | | | | |  |

Note: ^*^ P <0.05, ^* *^ P <0.01; ASC (Opt-out)：a specific constant item for opt-out; Ref: reference, which reflects a reference level in each attribute; *β* coefficient: which reflects the values of each attribute level and the horizontal regression coefficient; WTP: willingness to pay, which reflects residents’ willingness to pay for a certain screening program; 95% CI, 95% confidence interval.

**Supplementary Table S5.** Preference and WTP of residents in different genders

| **Attributes and levels** | **Male(n=315)** | | | **Female(n=611)** | | |
| --- | --- | --- | --- | --- | --- | --- |
|  | ***β* coefficient**  **(95% CI)** | ***Z* value** | **WTP**  **(95% CI)** | ***β* coefficient**  **(95% CI)** | ***Z* value** | **WTP**  **(95% CI)** |
| ASC(Opt-out) | -5.529  (-7.180,-3.878) | -6.560^**^ |  | -8.062  (-10.206,-5.918) | -7.370^**^ | - |
| Screening interval | | | | | | |
| Once in a lifetime (Ref) | | | | | | |
| Every year | -0.244  (1.035,1.646) | 8.590^**^ | 323.94  (236.02,411.87) | 1.119  (0.914,1.325) | 10.690^**^ | 252.74  (202.17,303.30) |
| Every 2 years | -0.244  (1.008,1.649) | 8.120^**^ | 321.12  (229.21,413.02) | 1.028  (0.793,1.262) | 8.590^**^ | 232.02  (175.84，288.20) |
| Every 5 years | -0.244  (0.643,1.313) | 5.720^**^ | 236.37  (158.55,314.20) | 0.964  (0.701,1.226) | 7.200^**^ | 217.58  (162.37,272.79) |
| Regular follow-up for precancerous lesions | | | | | | |
| Yes (Ref) | | | | | | |
| No | -0.244  (-0.413,-0.075) | -2.830^**^ | -58.99  (-100.27,-17.71) | -0.239  (-0.361,-0.116) | -3.820^**^ | -53.88  (-82.36,-25.39) |
| Morality reduction | | | | | | |
| 15% (Ref) | | | | | | |
| 30% | 0.103  (-0.179,0.386) | 0.720 | 25.00  (-44.11,94.11) | 0.072  (-0.150,0.294) | 0.630 | 16.26  (-34.19,66.71) |
| 45% | 0.333  (-0.006,0.672) | 1.920 | 80.40  (4.67,165.47) | 0.182  (-0.075,0.439) | 1.390 | 41.09  (-17.91,100.10) |
| 60% | 0.400  (0.120,0.679) | 2.800^**^ | 96.64  (26.57,166.70) | 0.086  (-0.111,0.283) | 0.860 | 19.48  (-25.37,64.34) |
| Screening technique | | | | | | |
| Endoscopy (Ref) | | | | | | |
| Painless (anesthesia) endoscopy | 2.734  (2.284,3.184) | 11.910^**^ | 660.62  (521.30,799.93) | 3.088  (2.730,3.446) | 16.910^**^ | 697.09  (603.12,791.06) |
| Out-of-pocket costs | -0.004  (-0.005,-0.003) | -9.210^**^ |  | -0.004  (-0.005,-0.004) | -13.170^**^ | - |

Note: ^*^ P <0.05, ^* *^ P <0.01; ASC (Opt-out): a specific constant item for opt-out; Ref: reference, which reflects a reference level in each attribute; *β* coefficient: which reflects the values of each attribute level and the horizontal regression coefficient; WTP: willingness to pay, which reflects residents’ willingness to pay for a certain screening program; 95% CI, 95% confidence interval.

**Supplementary Table S6.** Preference and WTP of residents with different annual household income

| **Attributes and levels** | **＜10000（n=434）** | | | **10000-29999（n=294）** | | | **≥30000（n=198）** | | |
| --- | --- | --- | --- | --- | --- | --- | --- | --- | --- |
|  | ***β* coefficient (95% CI)** | ***Z* value** | **WTP**  **(95% CI)** | ***β* coefficient (95% CI)** | ***Z* value** | **WTP**  **(95% CI)** | ***β* coefficient (95% CI)** | ***Z* value** | **WTP**  **(95% CI)** |
| ASC(Opt-out) | -7.501  (-9.792,5.210) | -6.420^**^ | *-* | -9.067  (-12.231,-5.903) | -5.620^**^ | - | -3.611  (-5.910,-1.312) | -3.080^**^ | - |
| Screening interval | | | | | | | | | |
| Once in a lifetime (Ref) | | | | | | | | | |
| Every year | 0.875  (0.618,1.133) | 6.660^**^ | 152.10  (106.16,198.05) | 1.166  (0.891,1.442) | 8.290^**^ | 315.01  (228.84,401.17) | 2.201  (1.711,2.691) | 8.810^**^ | 681.23  (457.06,905.40) |
| Every 2 years | 0.838  (0.539,1.137) | 5.500^**^ | 145.63  (92.88,198.39) | 1.143  (0.819,1.467) | 6.910^**^ | 309.83  (213.80,405.86) | 1.979  (1.486,2.473) | 7.860^**^ | 612.61  (396.92,828.30) |
| Every 5 years | 0.823  (0.501,1.145) | 5.010^**^ | 142.96  (90.18,195.73) | 0.925  (0.573,1.278) | 5.150^**^ | 251.22  (163.67,338.77) | 1.657  (1.066,2.249) | 5.490^**^ | 512.92  (328.21,697.63) |
| Regular follow-up for precancerous lesions | | | | | | | | | |
| Yes (Ref) | | | | | | | | | |
| No | -0.276  (-0.445,-0.107) | -3.190^**^ | -47.94  (-76.83,-19.05) | -0.341  (-0.499,-0.182) | -4.220^**^ | -90.97  (-137.89,-44.04) | -0.087  (-0.319,0.144) | -0.740 | -27.06  (-99.33,45.21) |
| Morality reduction | | | | | | | | | |
| 15% (Ref) | | | | | | | | | |
| 30% | 0.215  (-0.065,0.496) | 1.500 | 37.37  (-11.60,86.34) | 0.063  (-0.235,0.360) | 0.410 | 20.19  (-60.60,100.97) | -0.241  (-0.644,0.163) | -1.170 | -74.47  (-197.28,48.34) |
| 45% | 0.359  (0.038,0.680) | 2.190^*^ | 62.36  (5.97,118.76) | 0.224  (-0.124,0.573) | 1.260 | 61.57  (-35.03,158.18) | -0.022  (-0.513,0.468) | -0.090 | -6.91  (-158.26,144.44) |
| 60% | 0.049  (-0.212,0.311) | 0.370 | 8.57  (-36.95,54.09) | 0.360  (0.093,0.627) | 2.640^**^ | 99.18  (22.71,175.65) | 0.275  (-0.097,0.647) | 1.450 | 85.08  (-32.71,202.87) |
| Screening technique | | | | | | | | | |
| Endoscopy (Ref) | | | | | | | | | |
| Painless (anesthesia) endoscopy | 3.217  (2.724,3.710) | 12.790^**^ | 559.08  (473.70,644.47) | 2.707  (2.248,3.167) | 11.550^**^ | 732.14  (581.49,882.79) | 3.445  (2.708,4.182) | 9.170^**^ | 1066.26  (739.08,1393.43) |
| Out-of-pocket costs | -0.006  (-0.007,-0.005) | -10.900^**^ | - | -0.004  (-0.005,-0.003) | -8.890^**^ | - | -0.003  (-0.004,-0.002) | -6.170^**^ |  |

Note: ^*^ P <0.05, ^* *^ P <0.01; ASC (Opt-out)：a specific constant item for opt-out; Ref: reference, which reflects a reference level in each attribute; *β* coefficient: which reflects the values of each attribute level and the horizontal regression coefficient; WTP: willingness to pay, which reflects residents’ willingness to pay for a certain screening program; 95% CI, 95% confidence interval.
